# The frequency and relationship with vestibular function test results of positional preference in acute vestibular neuritis

**DOI:** 10.3389/fneur.2022.1033955

**Published:** 2022-10-20

**Authors:** Ji-Yun Park, Hyung Lee, Hyun Ah Kim

**Affiliations:** ^1^Department of Neurology, Ulsan University Hospital, University of Ulsan College of Medicine, Ulsan, South Korea; ^2^Department of Neurology, Keimyung University School of Medicine, Daegu, South Korea; ^3^Brain Research Institute, Keimyung University School of Medicine, Daegu, South Korea

**Keywords:** vestibular neuritis, positional preference, otolith function, spontaneous nystagmus, visual analog scale

## Abstract

**Objective:**

To assess the prevalence and relationship with vestibular function test results of positional preference in acute vestibular neuritis (VN).

**Methods:**

We prospectively recruited 33 patients with VN during the acute phase. We assessed the severity of vertigo with a visual analog scale (VAS) and the degree of spontaneous nystagmus (SN) during sitting, the head rolling to the affected, and the healthy side. Patients performed other vestibular function tests, including ocular and cervical vestibular evoked myogenic potential (VEMP), on the same day or the next day of VNG testing.

**Results:**

Twelve patients (12/33, 36%) with VN complained of more severe vertigo during lying on the affected side compared to the healthy side under visual fixation. Compared to patients without positional preference (without positional preference group), patients with positional preference (with positional preference group) showed a significantly higher VAS and maximal slow phase velocity (SPV) of SN at all positions except lying on the lesion side. However, there was no difference in the SPV gap between the two groups. 30% (10/33) of patients with VN complained of more severe vertigo while lying on the affected side compared to the healthy side without visual fixation. Maximal SPV of SN was not different between the two groups. There was no other significant difference in both canalith and otolith function test results between the two groups regardless of the visual fixation.

**Conclusions:**

One-third of patients with acute VN had more severe vertigo while lying on the affected side than in the supine position. The positional preference was not directly related to the SN intensity or VEMP results. The positional preference might reflect the otolith damage in the setting of activation of the sustained otolith system, not the transient otolithic system.

## Introduction

Vestibular neuritis (VN) is a common neuro-otologic syndrome characterized by acute prolonged vertigo (lasting several days), nausea, and vomiting without other accompanying neurologic or audiological symptoms or signs (1, 2). During the acute attack, patients usually lie with their eyes closed on a side with a healthy ear down (2). However, the frequency and mechanism of the positional preference in acute VN are unknown.

When a patient with acute VN sits up, especially with the head bending 30 degrees, the horizontal semicircular canal lies on the horizontal plane (3), and the effect of gravity on the semicircular canal and otolith organ on both sides would be the same. On the other hand, the difference of gravity between both vestibular organs would be the maximum on the lesion side up. The mechanism of this positional preference implies an interaction between inputs from the otoliths and the semicircular canals (4, 5). It has been suggested that the medial portion of the utricle may respond best to the pull of gravity (6). A previous study found that some of the patients with VN showed the inhibition of spontaneous nystagmus (SN) when they lay toward the healthy side and an increase of SN when they lay toward the lesion side (5). Authors suggested that an alteration in the influence of gravitation on the otolith organs can change SN in both a positive and a negative direction (5). Therefore, we assumed that the positional preference might be related to the utricular function tests such as subjective visual vertical (SVV) and ocular vestibular evoked myogenic potential (oVEMP) tests. We sought to investigate the frequency of positional preference and its relationship with semicircular and otolithic vestibular function tests in acute VN.

## Methods

We prospectively recruited patients with VN during the acute phase at Keimyung University Dongsan Hospital and Ulsan University Hospital from April 2019 to April 2020. All patients met the clinical diagnostic criteria for VN (1, 2, 7), including (1) sudden onset of prolonged vertigo (more than 1 day) within 1 week, (2) unidirectional spontaneous horizontal–torsional nystagmus, (3) the head-thrust test showed an ipsilateral deficit of the horizontal semicircular canal (gain < 0.8) or caloric asymmetry (>25%). Exclusion criteria were (1) the presence of auditory or other neurologic findings, (2) a previous history of neuro-otologic diseases, (3) abnormal audiogram or middle ear function, (4) acute lesion on diffusion-weighted brain MRI, (5) Severe cognitive impairment (Mini-mental state examination < 15). Diffusion-weighted MRI was performed on all patients to exclude a central lesion mimicking peripheral vestibulopathy.

**Figure 1:**
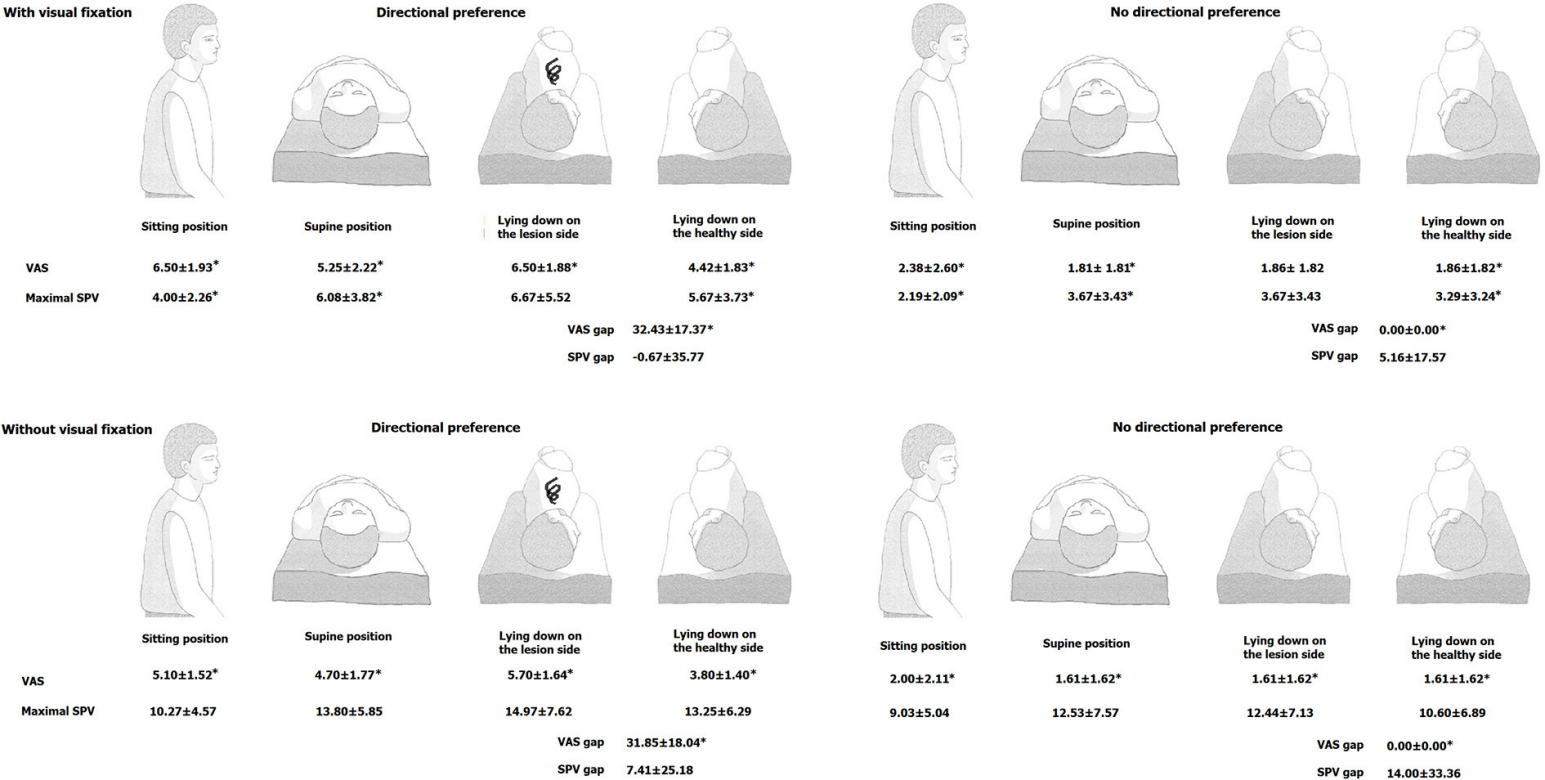
Comparison of the intensity of spontaneous nystagmus (maximal SPV) and vertigo severity (VAS) at each position between patients with vs. without positional preference. **(Upper)**: under the visual fixation, **(Lower)**: without visual fixation. SP, slow phase velocity; SN, spontaneous nystagmus; VAS, visual analog scale. *p < 0.05: with vs. without positional preference.

Videonystagmography (VNG) was used to record SN, head-shaking nystagmus (HSN), head-impulse test (HIT), and caloric responses. The maximal slow phase velocity (SPV) of SN was calculated during sitting, supine, lying on the affected side, and healthy side. We assessed the severity of vertigo with a visual analog scale (VAS) during each position (Figure 1). We defined the presence of positional preference if there is any difference in VAS between lying on the lesion and healthy sides. We checked maximal SPV and VAS with and without visual fixation. We also calculated the gap between maximal SPV and VAS between lying on the healthy and the lesion sides with the revised Jongkees difference equation (8).

SPV gap = (SPV_L– SPV_H)/SPV_L^*^100, where SPV_L is maximal SPV lying on the lesion side, and SPV_H is maximal SPV lying on the healthy side.

VAS gap = (VAS_L – VAS_H)/VAS_L^*^100, where VAS_L is VAS lying on the lesion side, and VAS_H is VAS lying on the healthy side.

Patients also performed oVEMP, cervical vestibular evoked myogenic potential (cVEMP), and SVV on the same day or the next day of VNG. We used a Nicolet Viking Select unit (Nicolet-Biomedical, Madison, WI, USA) to measure the surface electromyography (EMG) activity for cVEMP and oVEMP. A cVEMP was performed with the patient in a supine position, raising the head approximately 30° and rotating contralaterally to activate the sternocleidomastoid (SCM) muscles. The active electrode was placed over the belly of the contracted SCM after subtracting activity from a reference electrode located on the medial clavicle. A ground electrode was attached to the forehead. A short alternating tone burst (110 dB nHL; 500 Hz; ramp = 2 ms; plateau = 3 ms) was given at 2.1 Hz monoaurally using a headphone. To compare the amplitude of p13-n23 on the affected side (Aa) with that on the unaffected side (Au), the asymmetry ratio of each patient was calculated as (Aa – Au) / (Aa + Au) × 100 (9). We monitored the patients to ensure adequate levels of activation, enable fine adjustment of head position to match the EMG levels for each side, and allow measurement of background contraction levels and calculation of normalized amplitudes (9). Tests were considered abnormal if interaural differences in amplitude of p13 or n23 were outside the average value + 2 SDs (i.e., asymmetry of amplitude of cVEMPs > 28%) or no wave formation. Normative data were obtained from 36 age-matched healthy controls (14 men, mean age = 58.17 ± 10.59, *t*-test, *p* = 0.146) without a history of auditory or vestibular disorders.

To record oVEMP, the subject was supine on a bed, with the head supported on a pillow. An active electrode was placed 1 cm below the center of the lower eyelid, and the reference electrode was attached to the cheek 2 cm below the active electrode (10). The ground electrode was located on the forehead. During testing, the subject looked up approximately 25° above straight ahead and maintained a small fixation point ~60 cm from the eyes. oVEMPs were elicited by tapping the hairline using an electric reflex hammer (VIASYS Healthcare, CA, USA). Bilateral responses were simultaneously obtained whenever tapping stimuli were applied. Ocular VEMPs in response up to 60 stimuli were averaged for each test. The average latency of the initial negative peak (n10) and the n10 – p10 amplitude were analyzed. The interaural difference ratio of the amplitude of the ocular VEMPs was calculated as the interaural difference ratio (%) (Aa – Au) / (Aa + Au) × 100, where Aa and Au are the n10 – p10 amplitude on the affected and unaffected sides, respectively (10). Tests were considered abnormal if interaural differences in amplitude or latencies of n10 were outside the average value ± 2 SDs (i.e., asymmetry of amplitude of oVEMPs > 29%, amplitude of n10-p10 < 1.5 μV or latency of n10 > 21.5 ms) or no wave formation. Normative data were obtained from 28 age-matched healthy controls (19 men, mean age = 50.89 ± 11.54, *t*-test, *p* = 0.346) without a history of auditory or vestibular disorders.

The SVV tilt in the patients was defined as abnormal when the degree of SVV tilt exceeded the mean ± 2SD obtained from 80 normal controls (normal range: −2.0~2.0° for binocular viewing; the negative value indicates a counterclockwise tilt) (7). The detailed methods for recording SVV have been described previously (11).

Numerical parameters between patients with or without positional preference were calculated with a Mann-Whitney U test. A Fisher's exact test was performed for percentage comparison of nominal data, and Spearman's correlation was used for correlation analysis between parameters. Two-way ANOVA was used to determine both the independent effect of fixation and positional preference as well as to test whether there was a significant interaction between these variables.

All experiments complied with the tenets of the Declaration of Helsinki, and the study protocol was reviewed and approved by the Institutional Review Board of the School of Medicine at Keimyung University.

## Results

Thirty-three patients with acute VN were enrolled. The mean age was 53.9 ± 13.2 years. Approximately 76% of the patients were male, and 49% had VN on the right side. The time from onset to VNG was 53.0 ± 38.1 h.

### Vertigo severity and maximal SPV of SN at different positions with and without fixation

Under the visual fixation, the mean VAS during sitting position was 3.9 ± 3.1 and during supine position was 3.1 ± 2.6. Mean maximal SPV during sitting position was 2.8 ± 2.3 deg/sec and during supine position was 4.6 ± 3.8 deg/sec. The mean VAS during lying on the affected side was 3.6 ± 2.9, while lying on the healthy side was 2.8 ± 2.2. The mean maximal SPV during lying on the affected side was 4.8 ± 4.5 deg/sec, while lying on the healthy side was 4.2 ± 3.6 deg/sec. The mean VAS gap and SPV gap were 11.79 ± 18.83 and 3.10 ± 25.31, respectively.

When goggles were covered on the eyes (without visual fixation), the mean VAS during sitting position was 2.9 ± 2.4 and during supine position was 2.6 ± 2.2. Mean maximal SPV was 9.4 ± 4.9 deg/sec during sitting and 13.0 ± 7.1 deg/sec during supine position. The mean VAS during lying on the affected side was 2.9 ± 2.5, while lying on the healthy side was 2.3 ± 1.8. The mean maximal SPV during lying on the affected side was 13.3 ± 7.4 deg/sec, while lying on the healthy side was 11.6 ± 6.7 deg/sec. The mean VAS gap and SPV gap were 9.65 ± 17.67 and 12.00 ± 30.87, respectively.

Thirty-six percent (12/33) of the patients with VN complained of more severe vertigo during lying on the affected side compared to the healthy side (i.e., with directional preference group) under the visual fixation. The other patients reported no difference in vertigo severity while lying on the healthy side compared to the affected side (i.e., without positional preference group). Maximal SPVs during supine, sitting, and lying on the lesion side were significantly higher in the with positional preference group than those without positional preference group under the fixation. However, the mean SPV gaps between the two groups were no different (−0.67 ± 35.77 vs. 5.16 ± 17.57, respectively) (Figure 1 and Supplementary Table 1).

When goggles were covered on the eyes (without visual fixation), 30% (10/33) of patients with VN complained of more severe vertigo during lying on the affected side compared to the healthy side. There was no difference in maximal SPVs at any positions between the two groups without visual fixation. Maximal SPV and VAS at each position under two main factors (positional preference and visual fixation) with a two-way ANOVA showed no interaction between positional preference and visual fixation (Supplemental Table 1). There were significant positive correlations of VAS with maximal SPV at all positions under the visual fixation, but the VAS gap did not correlate with the SPV gap. There was no significant correlation of VAS with maximal SPV at all positions except lying on the lesion side (Table 1).

**Table 1:** Correlation analysis between the intensity of spontaneous nystagmus (maximal SPV) and vertigo severity (VAS) at each position.

	**Maximal SPV with fixation (deg/sec)**					**Maximal SPV without fixation (deg/sec)**				
**VAS**	**Sitting**	**Supine**	**Lying on the lesion side**	**Lying on the healthy side**	**SPV gap**	**Sitting**	**Supine**	**Lying on the lesion side**	**Lying on the healthy side**	**SPV gap**
Sitting	0.554^**^	0.487^**^	0.483^**^	0.592^**^	−0.149	0.320	0.283	0.354^*^	0.342	−0.030
Supine	0.568^**^	0.532^**^	0.503^**^	0.578^**^	−0.037	0.283	0.242	0.337	0.310	0.065
Lying on the lesion side	0.590^**^	0.552^**^	0.518^**^	0.602^**^	−0.039	0.278	0.249	0.330	0.342	0.002
Lying on the healthy side	0.508^**^	0.469^**^	0.478^**^	0.542^**^	−0.032	0.240	0.209	0.292	0.235	0.092
VAS gap	0.456^**^	0.377^*^	0.333	0.414^*^	−0.021	0.158	0.131	0.143	0.279	−0.136

Spearman's correlation coefficient.

* p < 0.05,

** p < 0.01.

SPV, slow phase velocity; VAS, visual analog scale.

### Vestibular function test results in patients with and without positional preference

There was no difference in age, lesion side, and time interval from symptom onset to the test between the two groups, regardless of the visual fixation. Female sex was significantly more frequent in patients with positional preference when the visual fixation was removed (13% vs. 50%, *p* = 0.036) (Table 2). The presence of positional preference was not different in patients with and without the abnormality of SVV, oVEMP, or cVEMP (Table 2). There was also no significant difference in canalith and otolith vestibular function test results between patients with and without positional preference (Table 3). The VAS and SPV gaps did not correlate with age, time interval from symptom onset to the test, or vestibular function test results, regardless of the visual fixation (Table 4).

**Table 2:** Sex, lesion side, otolithic function test results between patients with and without positional preference.

	**With visual fixation**			**Without visual fixation**		
	**No positional preference**	**With positional preference**	***p*-value**	**No positional preference**	**With positional preference**	***p*-value**
Sex (Female)	3/21 (14.3%)	5/12 (41.7%)	0.106	3/23 (13%)	5/10(50%)	0.036*
Lesion side (Lt)	13/21 (61.9%)	4/12 (33.3%)	0.157	14/23 (60.9%)	3/10 (30%)	0.141
Abnormal SVV	13/18 (72.2%)	9/11 (81.8%)	0.677	15/20 (75%)	7/9 (77.8%)	1.000
Abnormal oVEMP	11/21 (52.4%)	8/12 (66.7%)	0.486	12/23 (52.2%)	7/10 (70%)	0.455
Abnormal cVEMP	10/21 (47.6%)	3/12 (27.3%)	0.450	10/23 (43.5%)	3/9 (33.3%)	0.704

Fisher's exact test, ^*^p < 0.05.

SVV, subjective visual vertical; oVEMP, ocular vestibular evoked myogenic potential; cVEMP, cervical vestibular evoked myogenic potential.

**Table 3:** Vestibular function test results between patients with and without positional preference.

	**With visual fixation**					**Without visual fixation**				
	**No positional preference** **(*****n*** = **21)**		**With positional preference** **(*****n*** = **12)**			**No positional preference** **(*****n*** = **23)**		**With positional preference** **(*****n*** = **10)**		
	**Mean**	**SD**	**Mean**	**SD**	***p*-value**	**Mean**	**SD**	**Mean**	**SD**	***p*-value**
Age	55.95	13.12	50.42	13.24	0.410	54.87	13.83	51.80	12.16	0.681
Time interval from onset to test (hrs)	59.95	44.40	40.83	19.52	0.261	60.30	42.62	36.20	16.30	0.136
CP (%)	59.27	18.75	63.76	19.79	0.475	60.72	18.61	61.04	20.86	0.917
hHIT	0.37	0.15	0.33	0.12	0.370	0.35	0.15	0.35	0.12	0.903
pHIT	0.82	0.20	0.85	0.17	0.969	0.84	0.20	0.82	0.17	0.428
aHIT	0.60	0.15	0.65	0.10	0.258	0.60	0.15	0.66	0.09	0.230
SVV (°)	3.59	2.55	3.25	1.75	0.928	3.77	2.55	2.78	1.26	0.509
oVEMP (%)	50.21	37.71	49.16	38.09	1.000	50.86	38.25	47.50	36.78	0.935
n10 latency (ms)	6.93	6.52	9.33	6.78	0.270	7.17	6.91	9.28	6.00	0.312
n10-p10 amplitude (μV)	6.38	5.20	24.42	38.56	0.120	11.75	27.12	16.20	20.33	0.142
cVEMP (%)	44.50	39.02	26.56	28.68	0.184	41.71	38.31	29.41	30.91	0.358
HSN (deg/sec)	16.89	7.30	17.17	4.86	0.938	17.17	7.01	16.60	5.17	0.652

Mann-Whitney U-test.

SD, standard deviation; CP, canal paresis; hHIT, horizonal canal gain of head-impulse test; pHIT, posterior canal gain of head-impulse test; aHIT, anterior canal gain of head-impulse test; SVV, subjective visual vertical; oVEMP, ocular vestibular evoked myogenic potential; cVEMP, cervical vestibular evoked myogenic potential; HSN, head-shaking nystagmus.

**Table 4:** Correlation analysis of the gap of vertigo severity (VAS gap) and the gap of the intensity of spontaneous nystagmus (SPV gap) while lying on the lesion and healthy sides with vestibular function test results.

	**With fixation**				**Without fixation**			
	**SPV gap**		**VAS gap**		**SPV gap**		**VAS gap**	
	**r**	**p**	**r**	**p**	**r**	**p**	**r**	**p**
Age	−0.140	0.436	−0.139	0.441	0.089	0.623	−0.102	0.570
Time interval from onset to test (hrs)	−0.088	0.627	−0.171	0.341	0.127	0.480	−0.290	0.102
CP (%)	0.115	0.531	0.064	0.728	0.190	0.297	−0.030	0.872
hHIT	0.072	0.694	−0.170	0.352	−0.328	0.067	0.004	0.983
pHIT	−0.257	0.155	−0.033	0.858	0.024	0.895	−0.120	0.513
aHIT	0.003	0.987	0.158	0.389	0.023	0.899	0.230	0.206
SVV (°)	0.101	0.602	0.046	0.812	0.185	0.337	−0.129	0.504
oVEMP (%)	0.067	0.717	−0.010	0.957	−0.071	0.701	0.007	0.972
n10 latency (ms)	0.022	0.903	0.178	0.331	0.087	0.634	0.158	0.388
n10-p10 amplitude (μV)	0.010	0.958	0.309	0.085	−0.094	0.609	0.259	0.152
cVEMP (%)	0.023	0.901	−0.243	0.188	0.163	0.380	−0.133	0.475
HSN (deg/sec)	0.243	0.180	−0.023	0.901	0.000	0.999	−0.093	0.614

Spearman's correlation coefficient.

SPV, slow phase velocity; VAS, visual analog scale; CP, canal paresis; hHIT, horizonal canal gain of head-impulse test; pHIT, posterior canal gain of head-impulse test; aHIT, anterior canal gain of head-impulse test; SVV, subjective visual vertical; oVEMP, ocular vestibular evoked myogenic potential; cVEMP, cervical vestibular evoked myogenic potential; HSN, head-shaking nystagmus.

## Discussion

About 30% of patients with acute VN had positional preference, and all had more severe vertigo during lying on the affected side compared to the healthy side. Because SN intensity during lying on the lesion side was more prominent than lying on the healthy side (gravity-dependence) in the present study, we could assume that asymmetry of the vestibulo-ocular reflex may be reinforced during lying on the affected side and affect the SN intensity. However, the difference in vertigo severity between lying on the lesion and the healthy side did not relate to the gap in SN intensity. Moreover, SVV and VEMP test results were not associated with the gravity-dependence of SN intensity and positional preference.

Positional preference in VN has been thought to be due to the effect of gravity on the horizontal component of SN mediated by otolith-ocular reflex (5, 12). Because SN intensity in VN increased or inhibited according to head position, an alteration in the influence of gravitation on the otolith organs could change SN, which might be related to positional preference in patients with VN (13). As in the previous study (5, 13), patients with VN showed gravity-dependence of SN intensity in the present study. The mean maximal SPV was highest during lying on the affected side and lowest during the supine position. In the previous study, HSN in patients with unilateral VN is also modulated by the static attitude of the head and is more intense with the affected ear down (14). It has been suggested that an asymmetric suppression of vestibular nystagmus by the unilaterally impaired otolith organs could result in positional preference (5). Because the anterior semicircular canal is important in distinguishing between utricular activation by linear acceleration during lateral head tilt and by lateral head translation (15, 16), misinterpreted signals about head translation and head tilt in acute superior VN were suggested as a possible mechanism of positional preference. Another possible explanation for positional preference is linear vertigo. In patients with BPPV, the discrepancy between the internal representation of gravito-inertial acceleration provided by the otolith and the internal estimation of actual gravity direction increases an erroneous inertial acceleration (17–19). Similarly, we could assume that vestibular imbalance in VN could cause linear vertigo from transient central canal-otolithic perception change. If linear vertigo is additional to the original dizziness from the vestibular imbalance, it could cause positional preference in patients with VN.

Even though we could see gravity-dependent SN in patients with VN, the change of SN intensity during position change did not correlate with positional preference. Moreover, positional preference was not related to other semicircular or otolith function test results, including anterior canal gain of HIT, SVV, or VEMP in the present study. VEMP tests are clinical tests of transient otolith function evoked by brief sound and vibration stimuli and reflect the unilateral otolithic loss (20). In contrast, the sustained otolithic system concerned with signaling low-frequency gravitoinertial force stimuli could be tested with ocular counterrolling to roll-tilt or tests using linear translation. Although the SVV primarily reflects the asymmetry of utricular inputs between the sides of the vestibular system (21), the SVV most likely does not only reflect otolith function, but also depends directly and indirectly (*via* internal references) on the canal and proprioceptive function, and is affected by eye torsion upon the head roll (22). It seems that otolithic function test results in the present study could not simply reflect the stimulation of sustained otolithic system from lying on the lesion or healthy side.

Proprioception participates in the appreciation of body orientation and configuration, which is essential in vestibular rehabilitation in the acute stage (23). Asymmetric neck muscle proprioceptive signals from passive sustained head turning caused asymmetric functioning of the VOR in a previous study (24). Proprioception may also contribute to the generation of SVV change during head tilt (25, 26). There has been no previous study on the effect of lying to the healthy or lesion side on VOR. However, we could assume that the proprioceptive signal from lying also could affect the positional preference in acute VN.

Our study has some limitations. First, because the number of patients was relatively small, the possible contribution of the positional preference to the severity of vertigo and SN should be investigated again with larger sample size. Second, even though patients with VN usually prefer to lie in bed on the side with their healthy ears down and their eyes closed, they sometimes do not notice any difference in the severity of vertigo between lying on either side (2). We assessed the degree of vertigo during performing VNG, which could be different with positional preference in bed. Finally, some patients performed SVV, cVEMP, and oVEMP on the next day of VNG. We could not exclude the possibility of changing otolith functions within 1 day.

## Data availability statement

The raw data supporting the conclusions of this article will be made available by the authors, without undue reservation.

## Ethics statement

The studies involving human participants were reviewed and approved by Institutional Review Board of the School of Medicine at Keimyung University. The patients/participants provided their written informed consent to participate in this study.

## Author contributions

HK conducted the design and conceptualization of the study, interpretation of the data, and drafting and revising the manuscript. HL and J-YP interpreted the data and revised the manuscript. All authors contributed to the article and approved the submitted version.

## Funding

This work was supported by the research promoting grant from the Keimyung University Dongsan Medical Center in 2018.

## Conflict of interest

HL serves on the editorial boards of the Research in Vestibular Science, Frontiers in Neuro-otology, and Current Medical Imaging Review. The remaining authors declare that the research was conducted in the absence of any commercial or financial relationships that could be construed as a potential conflict of interest.

## Publisher's note

All claims expressed in this article are solely those of the authors and do not necessarily represent those of their affiliated organizations, or those of the publisher, the editors and the reviewers. Any product that may be evaluated in this article, or claim that may be made by its manufacturer, is not guaranteed or endorsed by the publisher.
