# Supplementary material for: The frequency and relationship with vestibular function test results of positional preference in acute vestibular neuritis
Source: Front Neurol. 2022 Oct 20;13:1033955. doi: 10.3389/fneur.2022.1033955 (PMC9642549; doi:10.3389/fneur.2022.1033955)
Supplement: Supplementary file 1 [file Table_1.docx]

**Supplemental Table 1**. Comparison of the intensity of spontaneous nystagmus (maximal SPV) and vertigo severity (VAS) at each position between patients with vs. without positional preference according to the visual fixation.

|  |  | With visual fixation | | | | | Without visual fixation | | | | | Two-way ANOVA | | |
| --- | --- | --- | --- | --- | --- | --- | --- | --- | --- | --- | --- | --- | --- | --- |
|  |  | No positional preference  (n=21) | | With positional preference  (n=12) | | Mann-Whitney U test | No positional preference  (n=23) | | With positional preference  (n=10) | | Mann-Whitney U test | p (Fixation) | p (Positional preference) | p (Interaction) |
|  |  | Mean | SD | Mean | SD | p-value | Mean | SD | Mean | SD | p-value |  |  |  |
| Maximal SPV of SN  (deg/sec) | Sitting | 2.19 | 2.09 | 4.00 | 2.26 | 0.010 | 9.03 | 5.04 | 10.27 | 4.57 | 0.398 | <.001 | 0.019 | 0.780 |
|  | Supine | 3.67 | 3.43 | 6.08 | 3.82 | 0.044 | 12.53 | 7.57 | 13.80 | 5.85 | 0.443 | <.001 | 0.063 | 0.701 |
|  | Lying on the lesion side | 3.67 | 3.43 | 6.67 | 5.52 | 0.065 | 12.44 | 7.13 | 14.97 | 7.62 | 0.421 | <.001 | 0.036 | 0.883 |
|  | Lying on the healthy side | 3.29 | 3.24 | 5.67 | 3.73 | 0.022 | 10.60 | 6.89 | 13.25 | 6.29 | 0.140 | <.001 | 0.032 | 0.925 |
|  | SPV gap | 5.16 | 17.57 | -0.67 | 35.77 | 0.709 | 14.00 | 33.36 | 7.41 | 25.18 | 0.510 | 0.223 | 0.400 | 0.960 |
| VAS | Sitting | 2.38 | 2.60 | 6.50 | 1.93 | <.001 | 2.00 | 2.11 | 5.10 | 1.52 | 0.001 | 0.182 | <.001 | 0.374 |
|  | Supine | 1.81 | 1.81 | 5.25 | 2.22 | <.001 | 1.61 | 1.62 | 4.70 | 1.77 | <.001 | 0.477 | <.001 | 0.714 |
|  | Lying on the lesion side | 1.86 | 1.82 | 6.50 | 1.88 | <.001 | 1.61 | 1.62 | 5.70 | 1.64 | <.001 | 0.313 | <.001 | 0.546 |
|  | Lying on the healthy side | 1.86 | 1.82 | 4.42 | 1.83 | 0.001 | 1.61 | 1.62 | 3.80 | 1.40 | 0.001 | 0.374 | <.001 | 0.679 |
|  | VAS gap | 0.00 | 0.00 | 32.43 | 17.37 | <.001 | 0.00 | 0.00 | 31.85 | 18.04 | <.001 | 0.937 | <.001 | 0.912 |

SPV=slow phase velocity; SN=spontaneous nystagmus; VAS=visual analog scale
